# PEGylated Graphene Quantum Dot Improved Cardiac Function in Rats with Myocardial Infarction: Morphological, Oxidative Stress, and Toxicological Evidences

**DOI:** 10.1155/2021/8569225

**Published:** 2021-11-20

**Authors:** Farzaneh Rostamzadeh, Mitra Shadkam-Farrokhi, Saeideh Jafarinejad-Farsangi, Hamid Najafipour, Zeinab Ansari-Asl, Mahboobeh Yeganeh-Hajahmadi

**Affiliations:** ^1^Physiology Research Center, Institute of Neuropharmacology, Kerman University of Medical Sciences, Kerman, Iran; ^2^Endocrinology and Metabolism Research Center, Institute of Basic and Clinical Physiology Sciences, Kerman University of Medical Sciences Kerman, Iran; ^3^Gastroenterology and Hepatology Research Center, Kerman University of Medical Sciences, Kerman, Iran; ^4^Cardiovascular Research Center, Institute of Basic and Clinical Physiology Sciences, Kerman University of Medical Sciences, Kerman, Iran; ^5^Department of Chemistry, Faculty of Science, Shahid Chamran University of Ahvaz, Ahvaz, Iran

## Abstract

**Introduction:**

The biocompatibility and potential application of graphene-based nanomaterials in biomedicine have been documented. The effects of polyethylene glycol-graphene quantum dots (GQDs-PEG) on cardiac function in rats with myocardial infarction (MI) were examined.

**Methods:**

Wistar rats were randomly assigned to two main groups, each consisting of sham-Veh., MI-Veh., and MI+GQDs-PEG at doses of 5, 10, and 20 mg/kg. MI was induced by the closure of the left anterior descending (LAD) coronary artery. After MI, GQDs-PEG were injected at different doses IP every other day for two weeks. In the end, hemodynamic and heart contractility indices were assessed. The levels of myocardial MDA (malondialdehyde), SOD (superoxide dismutase), GPX (glutathione peroxidase), and TAC (total antioxidant capacity) were measured by the ELISA method. The serum ALP, ALT, AST, creatinine, and urea levels were measured using the photometric method. The infarct size was assessed by TTC staining.

**Results:**

GQDs-PEG decreased the infarct size at doses of 10 and 20 mg/kg and recovered the MI-induced reductions of +dp/dt max and -dp/dt max in the study groups. GQDs-PEG normalized systolic blood pressure and left ventricular systolic pressure reduction at the dose of 20 mg/kg in the MI group. Heart SOD, GPX, and TAC increased in the GQDs-PEG 10 and 20 groups. Almost no signs of toxic effects due to GQDs-PEG administration were observed on the liver and kidneys.

**Conclusions:**

The results provided clear evidence that GQDs-PEG improve cardiac performance and hemodynamic parameters in rats with MI by reducing oxidative stress. GQDs-PEG is proposed as a therapeutic target for the treatment of MI.

## 1. Introduction

Myocardial infarction (MI) is one of the most serious cardiovascular diseases; it eventually leads to heart failure and is the leading cause of mortality and disability worldwide [1]. MI arises from the imbalance in the myocardial oxygen supply and demand [2]. The heart function deteriorates after myocardial infarction due to fibroblast proliferation, scar tissue formation, extracellular matrix degeneration, and heart remodeling [3]. Many therapeutic interventions have been discovered to prevent or reduce the remodeling processes after MI. Unfortunately, the prognosis and treatment of heart attacks are not currently efficient.

Nanomaterials, which are particles smaller than 100 nanometers (nm), are increasingly being used in the diagnosis and treatment of various diseases [4]. They are also used in targeted drug delivery and bioimaging. Graphene quantum dots (GQDs) are carbon-based zero-dimensional nanosheets with a lateral dimension of less than 100 nm with critical roles in biomedical research [5]. Because of their excellent biocompatibility, high water solubility, low cytotoxicity, and appropriate physicochemical properties, such as wide surface area [5, 6], GQDs have become suitable nanosheets used in therapeutic and diagnostic applications in neurological and inflammatory diseases and in cancer treatment [7, 8]. Due to the anionic group on their edges, immunological response to GQDs is uncommon [9], which further facilitates their biomedical application.

Reactive oxygen species (ROS) are among the initiators of heart injuries after MI and ischemia-reperfusion. ROS promote heart fibrosis by inducing fibroblast proliferation and increasing matrix metalloproteinase expression. [10]. Apoptosis and necrosis, hypertrophy, and heart remodeling have also been attributed to the oxidative/antioxidative imbalances following MI [10–12]. Atherosclerosis, one of the leading causes of MI, is characterized by an increase in oxidative stress. [13, 14]. It has been shown that GQDs could change the antioxidative/oxidative balances *in vivo* and *in vitro*. The ROS scavenging properties of GQDs decrease proinflammatory agents (interleukins) and subsequently improve colitis in experimental models. GQDs also increase the number of ROS scavenging macrophages, M2 marker-expressing cells [15]. The antioxidant activity of GQDs is due to the interaction of reactive oxygen radicals with electrons in the conjugated ring structures [8]. However, as far as we know, the direct effects of GQDs on antioxidant and oxidant levels have not been evaluated.

GQDs contain many reactive groups such as carboxyl, hydroxyl, and epoxy, which accumulate in different body compartments and organs [16, 17]. The addition of polyethylene glycol (PEG) to GQDs could prevent aggregation and amplification of colloidal forms of the nanoparticles [17–19]; increase their stability, half-life, and biocompatibility; and reduce their immunogenicity [17].

Regarding the high capability of graphenes to scavenge free radicals and the role of oxidative stress in the development of adverse consequences of MI, this study is aimed at evaluating the impacts of GQDs-PEG on the *outcomes* of MI induced by the occlusion of *the left anterior descending* coronary artery in rats.

## 2. Material and Methods

### 2.1. Materials

This study used sixty Wistar rats provided by the Kerman University of Medical Sciences animal house. Animals were kept in standard conditions with free access to tap water and regular feeding. Superoxide dismutase (SOD), glutathione peroxidase activity (GPX), and malondialdehyde (MDA) assay kits were purchased from ZellBio GmbH, Germany. Total antioxidant capacity (TAC) kits were obtained from Randox, United Kingdom. ALP, SGOT, SGPT, creatinine, and urea were measured via standard commercial kits (Parsazemon, Iran).

### 2.2. Methods

The experimental protocol was approved by the Ethics Committee of Kerman University of Medical Sciences (approval code: IR.KMU.REC.1398.019). Animals were randomly divided into two main groups, each consisting of five subgroups of sham-Veh. (phosphate-buffered saline, PBS), MI-Veh., and MI+GQDs-PEG 5, 10, and 20 mg/kg dissolved in PBS. GQD-PEG or Veh. was injected intraperitoneally every other day for two weeks after MI. The doses of GQD-PEG were used according to the study by Chong et al., where administration of 20 mg/kg of GQD-PEG for 14 days was found to be safe in *in vivo* and *in vitro* toxicity studies [20]. The hemodynamic indices, cardiac function indicators, and infarct size were measured in the first main groups. The samples from the second main group were used for molecular assessments.

### 2.3. Synthesis of GQDs and GQDs-PEG

GQDs were synthesized by the carbonization of citric acid [21]. Citric acid (1 g) was heated at 200°C in an oil bath until the color turned yellowish-orange.

After cooling at room temperature, the product was neutralized by NaOH. Finally, GQDs were purified from residual citric acid using a dialysis membrane (1 kDa) in distilled water for 48 h. After dialysis, the GQDs were freeze-dried and dissolved in EtOH (500 mg mL^−1^).

PEGylation of GQD was performed according to Chong et al. [20]. GQDs (0.2 mg/mL in phosphate buffer, 50 mM, pH 5.5) were activated with 1-ethyl-3-(3-dimethylamino propyl)-carbodiimide (EDC). PEG (2000 N, Sigma) was added to the GQD solution and stirred for 3 h. Finally, GQDs-PEG was concentrated by dialysis (3 kDa). GQDs and GQDs-PEG were characterized by ultraviolet-visible (UV-Vis) spectroscopy, transmission electron microscopy (TEM), DLS (dynamic light scattering) method, Fourier transform infrared spectroscopy (FT-IR), and nuclear magnetic resonance (NMR) spectroscopy.

### 2.4. Hemolysis Assay

Whole blood was collected in a K2EDTA anticoagulant tube and diluted in a calcium- and magnesium-free PBS solution at a ratio of 1 : 2. The solution was centrifuged at 500 × g for 10 min to isolate red blood cells (RBCs). The supernatant was removed gently, and the pellet was diluted with PBS (1 : 2 *v*/*v*) and centrifuged at 500 × g for 10 min. This process was repeated seven times. After the washing process, the pellet was diluted in a PBS solution (1 : 10). Then, 800 *μ*L of GQDs-PEG at concentrations of 1.5, 3, 4.5, and 6 mg/mL^−1^ was added to 200 *μ*L of diluted RBCs. All samples were incubated for 3 hours at 37°C and centrifuged for 3 minutes at 10016 × g. The proportion of hemolysis was estimated using the formula below:

(1) $$\begin{array}{c} \text{Hemolysis }\left(\%\right)=\frac{\text{Sample }{\text{abs}}_{540-655\text{ nm}}-\text{Negative Control a}{\text{bs}}_{540-655\text{ nm}}}{\text{Positive Control }{\text{abs}}_{540-655\text{ nm}}-\text{Negative Control a}{\text{bs}}_{540-655\text{ nm}}}. \end{array}$$

### 2.5. Induction of Myocardial Infarction

The left anterior descending (LAD) coronary artery was occluded for induction of MI, as previously explained [22]. After anesthesia with ketamine (80 mg/kg) and xylazine (10 mg/kg), the heart was exposed by an incision in the fourth left intercostal space under mechanical ventilation. The pericardium was opened, and the LAD was ligated 2 mm below its origin by a 6/0 silk suture. MI was verified by the pale appearance of the at-risk area and ST-segment elevation in ECG. In the sham group, as the control, the same anesthetic and surgical procedures were performed, except for the ligation of the LAD.

### 2.6. Recording of Hemodynamic Parameters and Cardiac Indices

Twenty-four hours after the last drug injection, rats were anesthetized with sodium thiopental (50 mg/kg). The blood pressure and heart rate were recorded by a catheter filled with heparin saline (10 units/mL) inserted in the right femoral artery. Another catheter was pushed into the left ventricle (LV) via the right carotid artery for measuring cardiac function indices [22]. The animal was ventilated through a tracheal cannula if necessary.

The arterial and ventricular cannulas were connected to pressure transducers and then to an 8-channel Powerlab system (ADInstruments, Australia). The hemodynamic parameters recorded were systolic blood pressure (SBP), diastolic blood pressure (DBP), and heart rate (HR). LV function indices were the left ventricular systolic pressure (LVSP), left ventricular end-diastolic pressure (LVEDP), and cardiac contractility indicators: maximum rate of rising in LV pressure (contraction velocity; +dp/dt max) and the maximum rate of decline in LV pressure (relaxation velocity; -dp/dt max).

### 2.7. Preparation of Heart and Serum Samples

The animals were euthanized under deep anesthesia, and their whole blood was collected. The clotted blood was centrifuged for 15 minutes at 3000 rpm. Then, the serum was separated and kept at -80°C. The removed hearts were rinsed with cold saline, and the left ventricle (LV) plus the septum was gently separated, weighed, and frozen at -80°C for measuring biochemical factors.

### 2.8. Measurement of the Heart Oxidant and Antioxidant Indices

The heart tissue samples from the peri-infarcted area were used to measure oxidants and antioxidant indices. To measure the SOD level, 100 mg of heart tissue was homogenized on ice in 1000 *μ*L of PBS (100 mM, pH = 7.4). The samples were then centrifuged at 5000 rpm for 20 min at 4°C. The supernatant (50 *μ*L) was used to measure the activity of SOD using the associated kit, according to the manufacturer's instructions.

Glutathione peroxidase activity was evaluated based on the colorimetric assay. 100 mg of the heart tissue was homogenized on ice in a 200 *μ*L assay buffer. The homogenate was centrifuged at 10000 × g for 15 min at 4°C, and the supernatant was used. Then, the GPX activity was measured using the associated assay kit.

100 *μ*L of homogenized tissue samples was used to measure MDA using thiobarbituric acid reaction, according to the kit's instructions. Tissue samples were lysed using 1.5% potassium chloride solution. The homogenate was centrifuged at 1200 rpm for 10 min. TAC levels were measured according to the instruction of the related kit.

### 2.9. Measuring Serum ALP, ALT, AST, Urea, and Creatinine

For evaluating the possible toxic effects of GQDs-PEG on the liver and kidneys, the serum levels of ALP, ALT, AST, urea, and creatinine were measured via the photometric method (Selectra E, Japan).

### 2.10. Measurement of Infarct Area Size

After recording hemodynamic and cardiac function parameters, the hearts were removed and rinsed in cold saline. The samples were kept at -20°C for 1 to 2 hours. Then, 3 mm slices were stained with triphenyl tetrazolium chloride 1% (TTC) in PBS for 20 min at 37°C. Finally, the slices were embedded in formaldehyde 10% for 10 min. The proportion of the infarcted area (pale) to the total LV area was measured by ImageJ software [23].

### 2.11. Statistical Analysis

The data were expressed as mean ± SEM. After verifying the normal distribution of data using the Shapiro-Wilk test, data were analyzed using one-way ANOVA, and in case of significance, pairwise comparisons were conducted by Tukey's post hoc test. An independent *t*-test was used for comparison between two groups. *P* values < 0.05 were considered to be statistically significant.

## 3. Results

### 3.1. Fabrication and Characterization of GQDs and GQDs-PEG

**Scheme 1:**
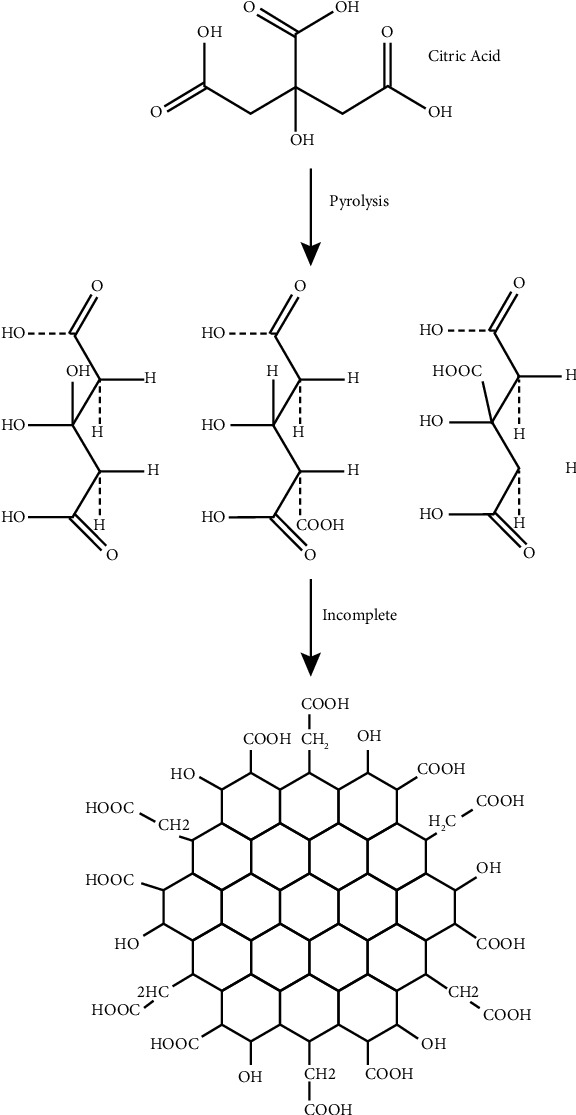
Reaction for the fabrication of GQDs from CA.

**Scheme 2:**
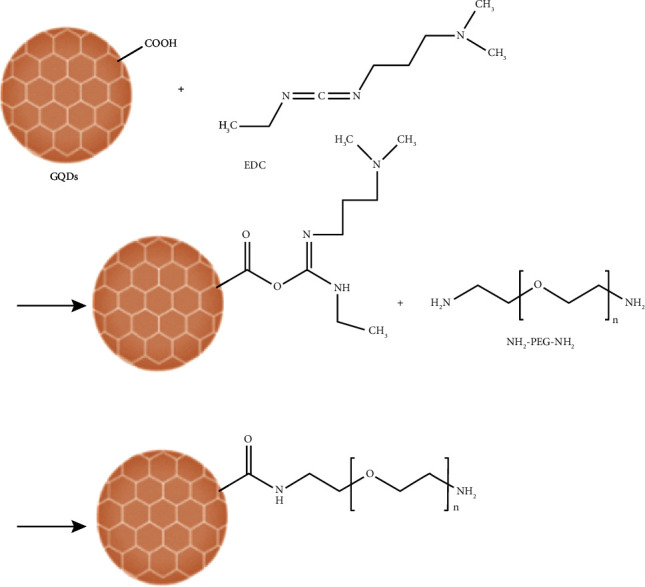
Reaction for the fabrication of GQDs-PEG.

According to Scheme 1, GQDs were synthesized through the pyrolysis of citric acid. The reaction for the fabrication of GQDs-PEG is shown in Scheme 2.

**Figure 1:**
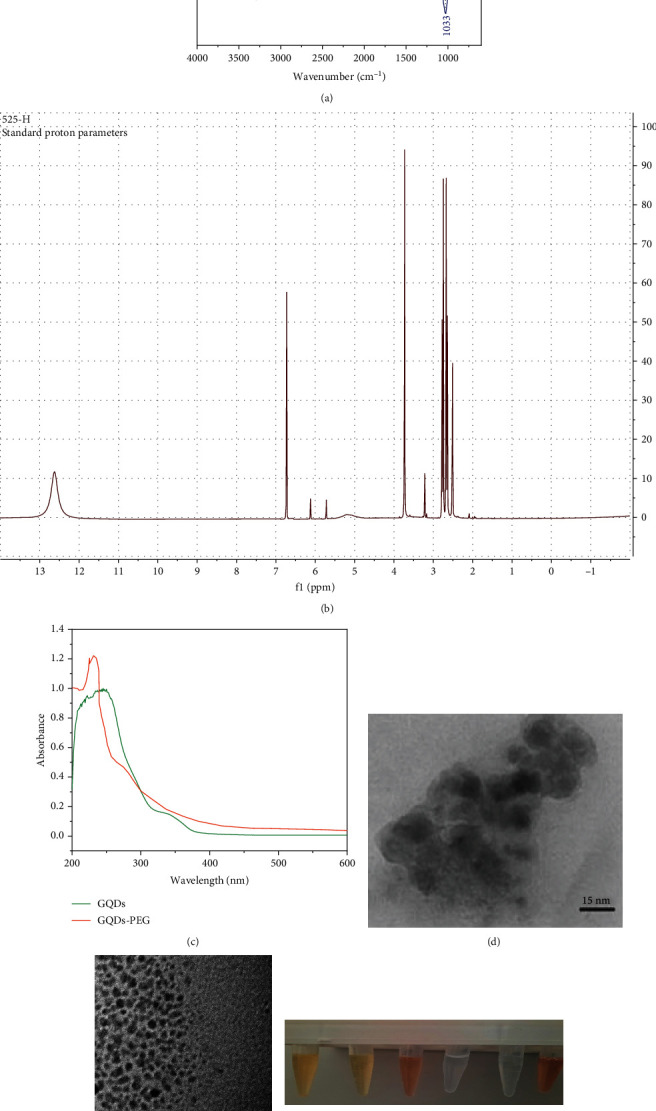
Characterization of GQD and GQDs-PEG. (a) FT-IR spectra of GQDs and GQDs-PEG. (b) ^1^HNMR spectrum of GQDs-PEG. (c) UV-Vis spectra of GQDs and GQDs-PEG. TEM image of (d) GQDs and (e) GQDs-PEG. (f) Dispersion stability of GQDs and GQDs-PEG in water, PBS, and serum after one month. Particle size distribution of the GQDs (g) and GQDs-PEG (h) by DLS analysis.

FT-IR spectra of the pure GQDs and GQDs-PEG are presented in Figure 1(a). In the FT-IR spectrum of GQDs, the absorption bands at 1033, 1228, 1616, and 1720 cm^−1^ are attributed to the C-O-C, C-OH, C=C, and C=O stretching vibrations, respectively. Other peaks can be referred to as C-H (2932 cm^−1^) and O-H (3334 cm^−1^). Incorporation of PEG and GQDs can be confirmed by the emergence characteristic PEG peaks at 1105 cm^−1^ (C-O-C groups) and 2879 cm^−1^ (C-H groups).

The as-obtained GQDs-PEG was further characterized by NMR (nuclear magnetic resonance) spectroscopy. HNMR spectrum of the GQDs-PEG in a DMSO-d6 solution is shown in Figure 1(b). In this spectrum, strong signals at *δ* = 2.5–5.7 ppm are related to protons near electronegative atoms such as N, O, or C=O groups. Furthermore, the singlet peak at *δ* = 6.1 ppm corresponds to the =CH proton. The presence of the PEG can be approved by the peaks exhibited at *δ* = 3.7 ppm, which are related to the CH_2_ (methane) protons.

The UV-Vis spectra of GQDs and GQDs-PEG are given in Figure 1(c). The GQDs and GQDs-PEG exhibit a characteristic absorption band around 235 nm due to the *π* − *π*^∗^ transitions. Since PEG has no absorption in the 200–600 nm wavelength region, the UV spectra of the GQDs and GQDs-PEG are almost similar. Additionally, the GQDs-PEG absorption band is more intense than the GQD peak, which may be related to defects in its structure.

Figures 1(d) and 1(e) show the transmission electron microscopy (TEM) images of GQDs and GQDs-PEG. It can be seen that the pure GQDs have a greater average size (15 nm) than the as-prepared GQDs-PEG (5 nm) owing to their tendency to aggregate.

We dissolved GQDs and GQDs-PEG in water, PBS, and serum for a month and then centrifuged solutions at 10000 × g for 5 min. The results in Figure 1(f) showed excellent stability of GQDs-PEG in water, PBS, and serum. We obtained a small pellet for GQDs in serum in comparing to GQDs-PEG. It seems that functionalization of GQDs with PEG has improved its biostability against salt- and protein-induced aggregations [18, 24].

The size distribution results of the GQDs and the GQDs-PEG in H_2_O solution (1 mg mL^−1^) are given in Figures 1(g) and 1(h). As can be seen, the mean particle size is around 5 nm and 10 nm for the GQDs and GQDs-PEG, respectively, and they exhibited a narrow particle size distribution that makes them proper for biological applications.

**Figure 2:**
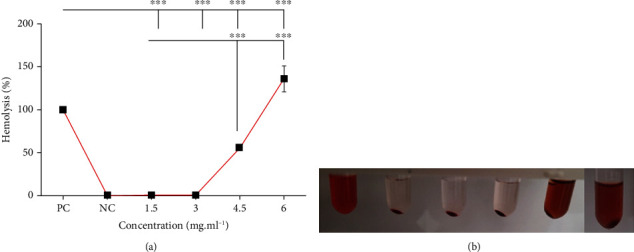
Hemolysis and disparity. (a) Cytotoxicity of GQDs-PEG in RBCs after 3 h exposure. (b) Image of RBCs exposed to different concentrations of GQDs-PEG. PC: positive control; NC: negative control; ^∗∗∗^*P* < 0.001.

RBC hemolysis assay is one of the important blood compatibility assessments for systemic administration of nanoparticles. The cytotoxicity of nanoparticles is measured by the amount of hemoglobin released from damaged RBCs [7]. According to our results, GQDs-PEG are not cytotoxic in RBCs at concentrations up to 3 mg mL^−1^. The hemolysis of RBCs at the concentration above 3 mg mL^−1^ increases and reaches about 50% at 4.5 mg mL^−1^. Since there is no significant hemolysis in RBCs after exposure to the high doses of 1500 and 3000 *μ*g mL^−1^, it seems that GQDs-PEG have good biocompatibility with RBCs (Figure 2).

### 3.2. The Effects of GQDs-PEG on Hemodynamic Parameters and Cardiac Indices

**Figure 3:**
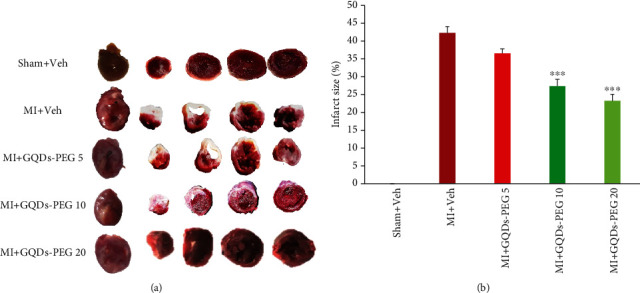
TTC staining revealed that GQDs-PEG 10 and 20 decrease infarct size. (a) The heart transverse sections in one animal of each group. (b) Quantification of infarct size. MI: myocardial infarction; GQDs-PEG: graphene quantum dots-polyethylene glycol; ^∗∗∗^*P* < 0.001 vs. MI, *n* = 5 in each group.

Body weight left ventricle weight/body weight ratio and lung weight/body weight ratio were not different among studied groups (Table 1). TTC staining indicated GQDs-PEG at the doses of 10 and 20 mg/kg significantly reduced the infarct size in the hearts (*P* < 0.001). GQDs-PEG at the 5 mg/kg dose had no significant effect on infarct size (Figures 3(a) and 3(b)).

**Figure 4:**
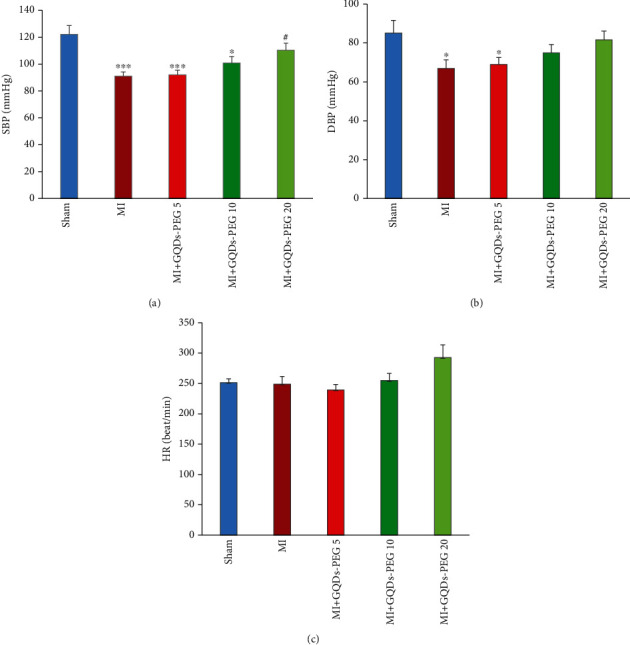
Effects of 14 days GQDs-PEG treatment at different doses on (a) SBP (systolic blood pressure), (b) DBP (diastolic blood pressure), and (c) HR (heart rate) in the studied groups. ^∗^*P* < 0.05, ^∗∗∗^*P* < 0.001 vs. sham; ^#^*P* < 0.05, ^###^*P* < 0.001 vs. the MI group. *n* = 7 in each group.

**Figure 5:**
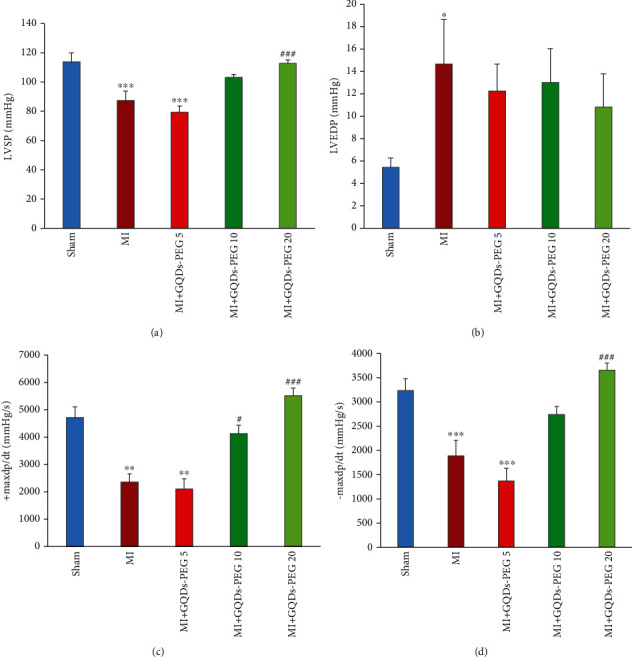
Effects of 14 days GQDs-PEG treatment at different doses on (a) LVSP (left ventricular systolic pressure), (b) LVEDP (left ventricular end-diastolic pressure), (c) +dp/dt max (maximum rate of increase in left ventricular pressure during systole), and (d) -dp/dt max (maximum rate of decrease in left ventricular pressure during diastole), in the studied groups. ^∗^*P* < 0.05, ^∗∗^*P* < 0.01, and ^∗∗∗^*P* < 0.001 vs. sham; ^#^*P* < 0.05, ^##^*P* < 0.01, and ^###^*P* < 0.001 vs. MI. *n* = 7 in each group.

SBP (*P* < 0.001) and DBP (*P* < 0.05) decreased in the MI group. These effects were inhibited by GQDs-PEG 20 (Figures 4(a) and 4(b)). The heart rate (HR) did not change among groups (Figure 4(c)). LVSP decreased in the MI group (*P* < 0.001). GQDs-PEG 10 nonsignificantly, and GQDs-PEG 20 (*P* < 0.001) significantly restored the reduction of LVSP (Figure 5(a)). LVEDP increased in the MI group. None of the doses of GQDs-PEG could correct the increase in LVEDP (Figure 5(b)).

+max dp/dt decreased significantly in the MI group (*P* < 0.001) and, GQDs-PEG at doses of 10 and 20 could cancel the impact of MI on this contractility index (*P* < 0.001) (Figure 5(c)). -max dp/dt also decreased in the MI group relative to the sham group (*P* < 0.001), and GQDs-PEG 20 could improve this relaxation index (*P* < 0.001) (Figure 5(d)). GQDs-PEG 5 had no significant effects on any of the above factors.

### 3.3. The Effect of GQDs-PEG on Antioxidant and Oxidant Factors

**Figure 6:**
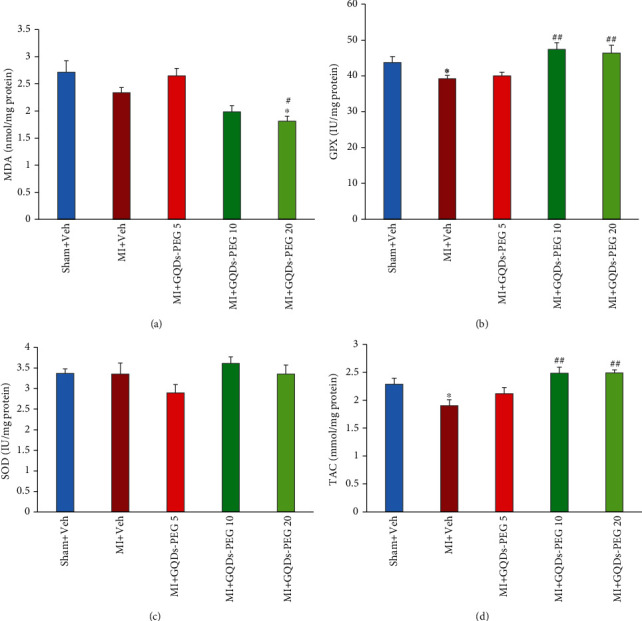
Effects of 14 days GQDs-PEG treatment at different doses on the left ventricle level of (a) MDA (malondialdehyde), (b) GPX (glutathione peroxidase), (c) SOD (superoxide dismutase), and (d) TAC (total antioxidant capacity) in rats with myocardial infarction in the studied groups. *n* = 7 in each group. ^∗∗^*P* < 0.01, ^∗∗∗^*P* < 0.001 vs. sham; ^##^*P* < 0.01, ^###^*P* < 0.001 vs. MI.

The impact of GQDs-PEG on the heart oxidative/antioxidative status was assessed in the samples of the border zone of the infarcted area of the left ventricle. MDA, as an oxidant index, was lower in the GQDs-PEG 20 group compared to other groups (*P* < 0.05) (Figure 6(a)). The levels of GPX and TAC decreased in the MI group (*P* < 0.05). GQDs-PEG at doses of 10 and 20 increased the levels of GPX and TAC in the hearts of animals with MI (*P* < 0.01) (Figure 6). SOD did not differ among groups.

### 3.4. Evaluation of the Toxicity of GQDs-*PEG*

**Figure 7:**
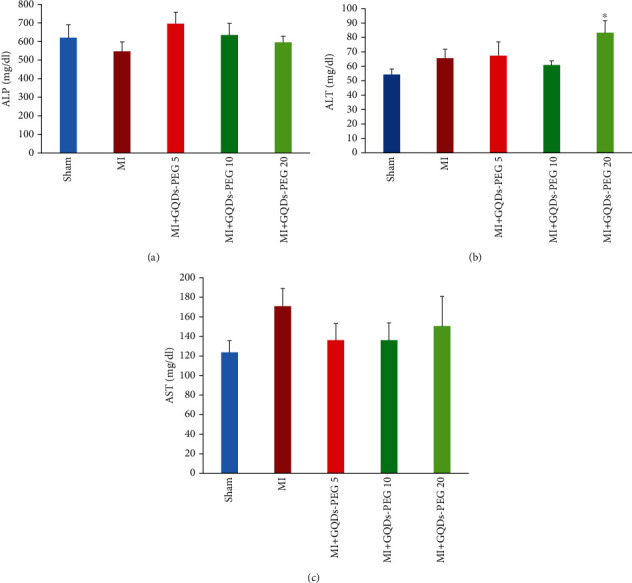
Effects of 14 days GQDs-PEG treatment at different doses on the serum levels of (a) ALP (alkaline phosphatase), (b) ALT (alanine aminotransferase), and (c) AST (aspartate aminotransferase) in the studied groups.. ^∗^*P* < 0.01 vs. sham, *n* = 7 in each group.

**Figure 8:**
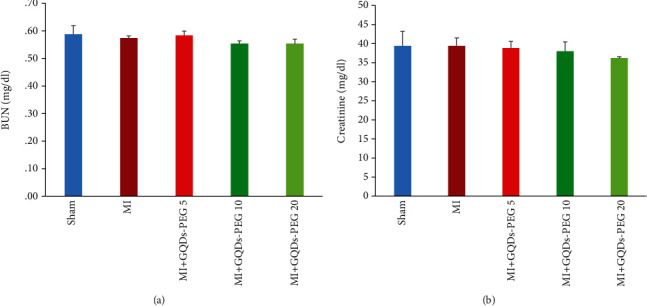
Effects of 14 days GQDs-PEG treatment at different doses on the serum levels of (a) urea and (b) creatinine in the studied groups. *n* = 7 in each group.

For evaluating the possible toxic effects of GQDs-PEG on the liver and kidneys, we measured alkaline phosphatase (ALP), aspartate aminotransferase (AST), alanine aminotransferase (ALT), urea, and creatinine in the serum. None of the liver function tests showed significant changes among the groups except for the effect of GQDs-PEG treatment at the dose of 20 mg/kg on ALP in the MI group (*P* < 0.05) (Figure 7). Blood urea nitrogen (BUN) and creatinine were not different among the groups (Figure 8).

## 4. Discussion

The study revealed that GQDs-PEG reduced infarct size, improved left ventricular function and cardiac contractility in the infarcted hearts, and normalized SBP and DBP in a dose-dependent manner. These beneficial effects were mediated by reducing oxidative stress in the heart, as GQDs-PEG increased myocardial GPX and TAC levels. The study also indicated that GQDs-PEG had no harmful effects on the kidneys at the doses used. In the case of the liver, only GQDs-PEG 20 increased ALT, one of the indicators of liver damage.

Graphene-based nanomaterials such as GQDs are used as carriers for other drugs such as anticancer medicines and for bioimaging due to their wide variety of functional groups (epoxy, hydroxyl, and carboxyl) and large surface area relative to their small size, as well as autofluorescence capability. There are several reports that directly describe their therapeutic effects. It was reported that graphene derivatives interact with immune cells such as macrophages [15] and T cells [25]. Therefore, GQDs have been suggested as therapeutic agents in inflammatory diseases [26]. GQDs also have protective impacts on Parkinson's disease through interaction with *α*-synuclein without complications [27, 28]. In line with the above studies, our results demonstrated that GQDs-PEG attenuated heart injuries and improved left ventricular contractility and cardiac function after MI. Our results also indicated that GQDs-PEG treatment had a greater effect on SBP than on DBP. This is the result of improvement in cardiac contractility because SBP is more readily affected by cardiac output than DBP [29].

Due to their reactive groups, one of the assumptions by which GQDs exert their beneficial effects is the regulation of ROS production. ROS production rises in patients after MI, especially after reperfusion, the phenomenon that causes progression of heart failure [30, 31]. Production of ROS in mitochondria after acute myocardial infarction damages cardiomyocytes and excessively increases the size of the infarcted area [32]. ROS stimulates ventricular hypertrophy and heart failure by induction of matrix metalloproteinase (MMPs) production and impairment of Ca+ hemostasis. Overexpression of superoxidase dismutase decreases infarct size in transgenic mice [32]. Our results suggested that GQDs-PEG attenuated the detrimental impacts of MI by reduction of oxidative stress. It was demonstrated that similar to catalase, graphene oxide quantum dots protect neurons against neurotoxicity induced by MPP+ [27].

The toxicity of nanoparticles has been one of the main concerns in their biomedical applications [33]. Many researchers have studied the therapeutic dosage and toxicity of graphene-based materials [20, 21]. Some of them have demonstrated that GQD had no detrimental impact on cells or organs. Fasbender et al. found that GQDs had no toxic effects on the viability of mononuclear blood cells [34]. Another study showed that the therapeutic and toxic effects of graphene-based materials depended on prescribed time and doses and the size of GQDs [35]. The present study's findings indicated that GQDs-PEG was safe at the doses used and increased ALT only at a dose of 20 mg/kg. The administration of GQDs in mice showed fast renal clearance, and it did not have toxicity detectable in changes in body weight, organs appearance, hematology tests, and blood cell analysis [20].

## 5. Conclusion

The results indicated that the administration of GQDs-PEG provided cardioprotection against MI injury in a dose-dependent manner. This effect is mediated by the reinforcement of the cardiac antioxidant defense system. The results suggested that GQDs-PEG may be considered potential therapeutic targets for treating MI injuries.

## Figures and Tables

**Table 1:** The body weight and heart and lung weight to body weight ratios in the study groups.

Groups variables	Sham-Veh.	MI-Veh.	MI+GQDs-PEG 5	MI+GQDs-PEG 10	MI+GQD-PEG 20
LVW/BW (mg/g)	2.21 ± 0.05	2.27 ± 0.08	2.12 ± 0.07	2.06 ± 0.12	2.07 ± 0.07
LW/BW (mg/g)	5.5 ± 0.16	6.1 ± 0.29	5.6 ± 0.23	5.9 ± 0.42	5.6 ± 0.33
BW (gr)	241 ± 12	242 ± 7	243.0 ± 6	233 ± 9	248 ± 7

LVW: left ventricle+septum weight; BW: body weight; LW: lung weight; MI: myocardial infarction; GQDs-PEG: graphene quantum dots-polyethylene glycol. Values are mean ± SEM. *n* = 7 in each group.

## Data Availability

Data are available on request.
